# The *Aspergillus flavus* Spermidine Synthase (*spds*) Gene, Is Required for Normal Development, Aflatoxin Production, and Pathogenesis During Infection of Maize Kernels

**DOI:** 10.3389/fpls.2018.00317

**Published:** 2018-03-20

**Authors:** Rajtilak Majumdar, Matt Lebar, Brian Mack, Rakesh Minocha, Subhash Minocha, Carol Carter-Wientjes, Christine Sickler, Kanniah Rajasekaran, Jeffrey W. Cary

**Affiliations:** ^1^Food and Feed Safety Research Unit, United States Department of Agriculture, Agricultural Research Service, Southern Regional Research Center, New Orleans, LA, United States; ^2^United States Department of Agriculture Forest Service, Northern Research Station, Durham, NH, United States; ^3^Department of Biological Sciences, University of New Hampshire, Durham, NH, United States

**Keywords:** *Aspergillus flavus*, spermidine synthase, polyamines, aflatoxin, mycotoxin, polyamine uptake, amino acids

## Abstract

*Aspergillus flavus* is a soil-borne saprophyte and an opportunistic pathogen of both humans and plants. This fungus not only causes disease in important food and feed crops such as maize, peanut, cottonseed, and tree nuts but also produces the toxic and carcinogenic secondary metabolites (SMs) known as aflatoxins. Polyamines (PAs) are ubiquitous polycations that influence normal growth, development, and stress responses in living organisms and have been shown to play a significant role in fungal pathogenesis. Biosynthesis of spermidine (Spd) is critical for cell growth as it is required for hypusination-mediated activation of eukaryotic translation initiation factor 5A (eIF5A), and other biochemical functions. The tri-amine Spd is synthesized from the diamine putrescine (Put) by the enzyme spermidine synthase (Spds). Inactivation of *spds* resulted in a total loss of growth and sporulation *in vitro* which could be partially restored by addition of exogenous Spd. Complementation of the *Δspds* mutant with a wild type (WT) *A. flavus spds* gene restored the WT phenotype. In WT *A. flavus*, exogenous supply of Spd (*in vitro*) significantly increased the production of sclerotia and SMs. Infection of maize kernels with the *Δspds* mutant resulted in a significant reduction in fungal growth, sporulation, and aflatoxin production compared to controls. Quantitative PCR of *Δspds* mutant infected seeds showed down-regulation of aflatoxin biosynthetic genes in the mutant compared to WT *A. flavus* infected seeds. Expression analyses of PA metabolism/transport genes during *A. flavus*-maize interaction showed significant increase in the expression of arginine decarboxylase (*Adc*) and *S*-adenosylmethionine decarboxylase (*Samdc*) genes in the maize host and PA uptake transporters in the fungus. The results presented here demonstrate that Spd biosynthesis is critical for normal development and pathogenesis of *A. flavus* and pre-treatment of a *Δspds* mutant with Spd or Spd uptake from the host plant, are insufficient to restore WT levels of pathogenesis and aflatoxin production during seed infection. The data presented here suggest that future studies targeting spermidine biosynthesis in *A. flavus*, using RNA interference-based host-induced gene silencing approaches, may be an effective strategy to reduce aflatoxin contamination in maize and possibly in other susceptible crops.

## Introduction

Mycotoxin contamination in food and feed crops is a global problem. Exposure to mycotoxins primarily occurs through the consumption of contaminated seeds/edible plant parts by humans and livestock. The majority of mycotoxin contamination in crop plants comes from the fungal genera, *Aspergillus*, *Fusarium*, and *Penicillium*, with *Aspergillus* causing the greatest adverse economic and health impacts (Ismaiel and Papenbrock, 2015; Mitchell et al., 2016; Umesha et al., 2016). Maize is a major food and feed crop grown worldwide and is susceptible to *Aspergillus flavus* infection and subsequent contamination with aflatoxins and other toxic SMs thus posing a serious threat to food security worldwide (Mitchell et al., 2016; Umesha et al., 2016). Contamination of crops with aflatoxins has been shown to be intensified during episodes of drought (Kebede et al., 2012; Fountain et al., 2014). Increases in aflatoxin contamination in maize during episodes of drought and heat stress is believed to be due to the response of *A. flavus* to increased oxidative stress as well as impairment of host plant defense responses (Fountain et al., 2014, 2016). Although drought tolerance alone does not necessarily result in increased aflatoxin resistance in maize, drought tolerance accompanied with aflatoxin resistance would be ideal in reducing aflatoxin accumulation in maize during drought (Hamidou et al., 2014; Farfan et al., 2015; Fountain et al., 2015). Aflatoxin contamination in maize results in economic losses of almost $700 million/year in the U.S. based on a study conducted in 2013 (Mitchell et al., 2016). Based on global climate change predictions, it is estimated that losses resulting from aflatoxin contamination of maize could be as high as US$1.68 billion/year in the United States. (Mitchell et al., 2016). Given the adverse impacts of aflatoxins to humans and livestock, pre-harvest control might be the best way to prevent aflatoxin contamination in food and feed commodities. Methods for pre-harvest control of aflatoxin contamination in maize include, (i) introduction of resistance genes against *A. flavus*; (ii) rational design of inhibitors of fungal biochemical pathways/enzymes required for aflatoxin production; (iii) biological control; and (iv) use of modern functional genomics tools to inhibit expression of fungal genes critical to maize colonization and aflatoxin production.

**FIGURE 1:**
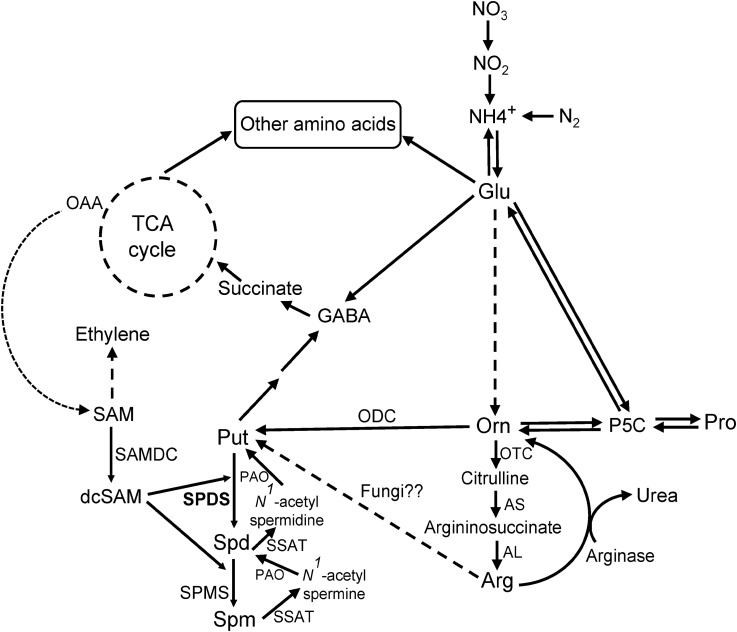
Polyamine (PA) pathway. Overview of the PA biosynthetic pathway (modified from Majumdar et al., 2015) in connection with amino acids (AAs) and tricarboxylic (TCA) cycle metabolites in plants and fungi. Dashed arrows indicate multiple steps. Abbreviations of enzymes, with EC numbers: AL, argininosuccinate lyase (EC 4.3.2.1); arginase (EC 3.5.3.1); AS, argininosuccinate synthase (EC 6.3.4.5); ODC, ornithine decarboxylase (EC 4.1.1.17); OTC, ornithine transcarbamylase (EC 2.1.3.3); PAO, polyamine oxidase (EC 1.5.3.11); SAMDC, *S*-adenosylmethionine decarboxylase (EC 4.1.1.50); SPDS, spermidine synthase (EC 2.5.1.16); SPMS, spermine synthase (EC 2.5.1.22); SSAT, Spd/Spm *N*^1^-acetyl-transferases (EC 2.3.1.57). Other abbreviations: dcSAM, decarboxylated *S*-adenosylmethionine; GABA, γ-aminobutyric acid; Glu, glutamate; OAA, oxaloacetic acid; P5C, Δ^1^-pyrroline-5-carboxylate; Pro, proline; Put, putrescine; SAM, *S*-adenosylmethionine; Spd, spermidine; Spm, spermine; TCA, tricarboxylic acid.

A number of candidate fungal genes have been targeted to control plant disease including PA metabolic genes (Valdés-Santiago et al., 2012; Liao et al., 2015; Jasso-Robles et al., 2016). PAs are ubiquitous, small aliphatic, poly-cationic, biogenic amines of wide-spread occurrence throughout all life forms. They are involved in plethora of cellular processes in living organisms including interactions with DNA (replication, transcription, and translation), transporter function, scavenging of oxidative stress molecules, growth and development, and stress response (reviewed in Valdés-Santiago et al., 2012; Valdés-Santiago and Ruiz-Herrera, 2013; Minocha et al., 2014; Miller-Fleming et al., 2015; Masson et al., 2017). The three predominant PAs widely found in living organisms are, Put (diamine), Spd (tri-amine), and Spm (tetramine). The diamine Put is produced by Odc (E.C.4.1.1.17) from Orn and/or by Adc (EC 4.1.1.19) from Arg. Higher PAs, Spd, and Spm are synthesized by the action of Spds (E.C.2.5.1.16) and Spms (E.C.2.5.1.22) from Put and Spm, respectively (reviewed in Shao et al., 2012). Both reactions require decarboxylated *S*-adenosylmethionine (dcSAM) that is produced by Samdc (E.C.4.1.1.50). Spm and Spd on the other hand can be back-converted to Spd and Put, respectively, by Spd/Spm N1-acetyltransferase (Sat; E.C.2.3.1.57 – mostly in animals) and Pao (E.C.1.5.3.11 – animals and plants). The predominant PAs that are often found in fungi are Put and Spd, while some fungal genera might lack Spm (reviewed in Valdés-Santiago et al., 2012). Given the requirement of PAs to maintain normal growth, development, and pathogenesis, the PA biosynthetic pathway (**Figure 1**) has often been a target to restrict fungal pathogenesis in both plants and animals (reviewed in Valdés-Santiago et al., 2012). In fungi, while Put is associated with hyphal growth, Spd has been implicated in cellular events associated with cell division, sporulation, and mycotoxin production. In the model fungus *Aspergillus nidulans* (*A. nidulans*), inactivation of *spds* altered fungal growth in the mutant (an auxotroph for Spd) and reduced sterigmatocystin production *in vitro* (Jin et al., 2002). In the human pathogenic fungus *Penicillium marneffei* (*P. marneffei*), impairment of Spd biosynthesis in a *samdc* (*sadA*) mutant reduced growth, conidiogenesis, spore germination, and temperature-dependent dimorphic transition (Kummasook et al., 2013). Exogenous supply of Spd to the *P. marneffei sadA* mutant could restore the WT phenotype. In wheat, early activation of the PA biosynthetic pathway has been reported in response to Fusarium head blight and PA pathway intermediates have been correlated with the production of deoxynivalenol (DON; Gardiner et al., 2009, 2010). In fact, Put activated the production of DON by regulating the expression of its biosynthetic genes (Gardiner et al., 2009).

Polyamines are common to both plants and fungal pathogens, and their metabolism can play significant roles in host defense as well as successful pathogenesis and mycotoxin production during compatible host-pathogen interactions (Gardiner et al., 2009, 2010; Valdés-Santiago and Ruiz-Herrera, 2013; reviewed in Takahashi, 2016). It was demonstrated that the fungus *Tapesia yallundae*, rendered avirulent due to inactivation of the *odc* gene, had virulence restored upon re-introduction back into plants, possibly due to the uptake of PAs from the host thus compensating for PA depletion in the pathogen (Mueller et al., 2001). One of the important genes in higher PA biosynthesis is *spds*, whose product Spd, plays a critical role in mRNA translation due to its requirement for hypusination-mediated activation of eukaryotic translation initiation factor 5A (eIF5A) in all organisms, including fungi (Martinez-Rocha et al., 2016). The current study was undertaken to investigate if the *A. flavus spds* (AFLA_017920) gene could be a suitable target to reduce fungal pathogenesis during maize seed infection. Our results show that inactivation of *spds* in *A. flavus* significantly reduced fungal infection, sporulation and aflatoxin production *in vitro* and during maize seed infection.

## Materials and Methods

### Fungal Strains, Media, and Growth Conditions

*Aspergillus flavus* CA14 (*Δku70*, *ΔpyrG*, *ΔniaD*) was used as host for transformation. Unless otherwise stated, CA14 pyrG-1, transformed with plasmid pPG2.8 (Cary et al., 2015) carrying the *A. parasiticus pyrG* gene was used as control for all experiments. A WT *A. flavus* 70 (AF70), capable of producing significantly higher levels of aflatoxins and sclerotia than CA14, was used to study the effect of PAs on SM and sclerotia production. A WT CA14 was used as the control for maize kernel infection studies. Fungal strains were cultured on Czapek-Dox (CZ) medium (Difco, BD). The medium was supplemented with Spd (0.5 or 0.25 mM) or Spm (0.2 or 0.1 mM) [both Spd and Spm were purchased from Sigma-Aldrich, St. Louis, MO, United States] as required. As CZ medium is not conducive for aflatoxin production, A&M medium (Mateles and Adye, 1965) supplemented with Spd and Spm was used to study the effects of PAs on aflatoxin production. Solid media were prepared by adding 15 g.L^-1^ of agar. Fungal cultures were grown in light or dark at 30°C.

For maize kernel inoculation studies, an aflatoxin-producing *A. flavus* CA14 WT strain was obtained from the SRRC fungal collection (SRRC 1436; USDA Agricultural Research Service, New Orleans, LA, United States). The fungal strain was grown on CZ agar medium for 7 days at 30°C with illumination. Conidia were harvested by flooding each plate with 20 ml of 0.02% (v/v) sterile Triton X-100 solution and gently dislodging conidia from the surface mycelia using a sterile scraper. Conidial suspensions were adjusted to 4 × 10^6^ spores/ml prior to inoculation of kernels.

### Generation of Deletion and Complementation *spds* Strains

The *spds* (AFLA_017920) deletion cassette was constructed by fusion PCR as described by Szewczyk et al. (2006). The 5′ and 3′ regions flanking the *spds* gene were PCR amplified from *A. flavus* CA14 genomic DNA using primer sets *Spds_*5F*/*Spds_5R and *Spds_*3F*/Spds_*3R, respectively (Supplementary Table S1). The middle fragment containing the *pyrG* marker gene was PCR amplified from the cDNA of *A. parasiticus* (BN9 strain), using the primers *pyrG*_F and *pyrG*_R (Supplementary Table S1). The three PCR fragments were then fused together through PCR using the nested primer pair *Spds*_nest-F and *Spds*_nest-R. The final PCR product (3739 bp) was used for polyethylene glycol-mediated transformation of *A. flavus* CA14 protoplasts as described by Cary et al. (2006). A number of putative *Δspds* transformants (*Δku70*, *ΔniaD, pyrG+*) were isolated and analyzed by PCR. Replacement of the *spds* coding region with *pyrG* selectable marker was confirmed through PCR using the primer pair *Spds*_5F and *pyrG_R* (Supplementary Figure S1). CA14 transformed with plasmid pPG2.8 (Cary et al., 2015) carrying the *A. parasiticus pyrG* gene was used as an isogenic control (referred to as the control).

To genetically complement the CA14 *Δspds* mutant, the *spds* gene region including 615 bp of the 5′ UTR and 286 bp of the 3′ UTR was PCR amplified from WT CA14 gDNA using prom_F2 and term_R primers. The *A. parasiticus* (BN9 strain) *pyrG* selectable marker gene was PCR amplified using primer pair *pyrG*_F1 and *pyrG*_R1, and was fused to the 2.6 kb *spds* PCR product through overlap fusion PCR using primer pairs prom_nest-F and *pyrG*_nest-R, that generated a 4.1 kb PCR product. The final PCR product (*spds*-*pyrG*, 4.1 kb) was used for polyethylene glycol-mediated transformation of *Δspds* CA14 (*Δku70, ΔniaD, pyrG+*) protoplasts. To select genetically complemented *Δspds* transformants (*Δspds^C^*, *Δku70, ΔniaD, pyrG+*), protoplasts (post-transformation) were plated onto regeneration medium (Cary et al., 2006) without Spd. Replacement of the *Δspds* coding region with the *spds*-*pyrG* complementation PCR product was confirmed through PCR using the primer pair 017920prom_nest-F and *pyrG*_nest-R (Supplementary Figure S2). PCR amplifications for creating the *Δspds* knockout construct were performed using ExTaq HS polymerase (Takara Bio, Inc., Mountain View, CA, United States), while the *spds*-*pyrG* complementation construct was generated using Phusion polymerase (New England BioLabs, Ipswich, MA, United States). The primers used in construction of the knockout mutant and complementation strain are listed in Supplementary Table S1.

### Nucleic Acid Isolation and Analysis

Fungal genomic DNA was extracted from mycelia following 24 h incubation with shaking (250 rpm) at 30°C in CZ broth using a MasterPure Yeast DNA Purification Kit (Epicentre, Madison, WI, United States) according to the manufacturer’s instructions. To confirm the successful integration of either knockout or complementation gene cassettes, genomic DNA isolated from transformants was PCR amplified using ExTaq HS polymerase and specific primer pairs listed in Supplementary Table S1.

### Morphological Analysis

Conidia (10^6^ spores/ml) of the CA14 control and *Δspds* mutant strains were used to inoculate CZ agar plates with or without Spd supplementation and grown under illumination at 30°C. Six-millimeter diameter cores were collected from the center of each colony (from three replicate plates) at 5 days post-inoculation (dpi). The cores were homogenized, and conidia were counted using a Hemocytometer (Hausser Scientific, Horsham, PA, United States) and a Leitz Laborlux S bright-field microscope (Leica Microsystems, Inc., Buffalo Grove, IL, United States).

The AF70 strain was used to study the effects of PAs on sclerotia and SM production. Conidia (10^6^ spores/ml) of AF70 were point inoculated (2 μl) at the center of CZ agar plates supplemented with Spd (0.25 and 0.50 mM) or Spm (0.1 and 0.2 mM) along with control plates (CZ only) in duplicate. The culture plates were grown in the dark at 30°C. After 14 days, sclerotia were counted using a Leitz Laborlux S bright-field microscope under 10× optical lens. Plate images were captured using a PowerShot SD790 IS camera (Canon USA Inc., Melville, NY, United States).

### Maize Kernel Inoculation and Incubation

Undamaged and roughly uniform sized maize (*Zea mays* var. B73) kernels were randomly assigned and processed according to a kernel screening assay (KSA; Rajasekaran et al., 2013). All kernels were surface sterilized using 70% ethanol, air dried, and stored under sterile conditions. Kernels were inoculated by immersion into a suspension of 4 × 10^6^ spores/ml of the CA14 WT (grown on CZ agar medium), CA14 WT w/Spd (CZ agar medium + 0.5 mM Spd), and *Δspds* w/Spd (CZ agar medium with ammonium sulfate + 0.5 mM Spd) strains followed by stirring for 3 min. Following removal of excess inoculum, the kernels were transferred to plastic caps that were placed in trays with a sheet of 3 MM paper on the bottom. The paper was saturated with sterile ddH_2_O and the tray covered with a lid. The kernels were incubated under high RH (>90%) at 31°C in the dark for 8 days. The filter papers inside the trays were kept moist by adding sterile water when needed during this incubation period.

### Quantification of Polyamines and Amino Acids

Freeze dried mycelia were subjected to three cycles of freezing (-20°C) and thawing (room temperature) in 5% PCA. After the final thaw, samples were vortexed for 2 min and centrifuged for 8 min at 14,000 ×*g*. PAs and AAs were simultaneously dansylated and quantified using an HPLC method from Minocha and Long (2004) with following modifications. Samples were incubated at 60°C for 30 min, cooled for 3 min and then microfuged at 14,000 ×*g* for 30 s. The reaction was terminated by the addition of 45 μl of glacial acetic acid. Sample tubes were kept open for 3 min under a flow hood to allow CO_2_ bubbles to escape. Acetone used to dissolve dansyl chloride was evaporated using a SpeedVac Evaporator (Savant, Farmingdale, NY, United States) for 5 min. Finally, 1735 μl of filtered HPLC grade methanol was added to all tubes bringing the total volume to 2 ml. PAs and AAs were analyzed by HPLC method according to Minocha and Long (2004). The data were processed using Perkin Elmer TotalChrom software (version 6.2.1).

### Secondary Metabolite Analysis

Maize kernels (∼20–70 mg) inoculated with WT CA14 and *Δspds* mutant strains were homogenized and extracted in 1 ml of ethyl acetate/acetone (1:1)/0.1% formic acid solution at room temperature for 24 h. The extracts were filtered through cotton plugs and the filtrates were concentrated under N to dryness. Each extract was re-dissolved in acetonitrile (1 mg/ml), filtered through a 0.22 μm Spin-X centrifuge tube filter, and analyzed using a Waters ACQUITY UPLC system (40% methanol in water, BEH C18 1.7 μm, 2.1 mm × 50 mm column) using fluorescence detection (Ex = 365 nm, Em = 440 nm). Samples were diluted to 10-fold if the aflatoxin signal saturated the detector. Analytical standards (Sigma-Aldrich, St. Louis, MO, United States) were used to identify and quantify aflatoxins: aflatoxin B1 (AFB1); aflatoxin B2 (AFB2). Aflatoxin content was expressed in ng/mg fresh weight of homogenized kernels.

For analysis of other SMs, AF70 was grown on CZ agar with ammonium sulfate (supplemented with Spd at 0.5 and 0.25 mM or Spm at 0.2 and 0.1 mM) medium at 30°C in the dark for 14 days along with controls with no PAs. Fungal cultures were lyophilized and then extracted with ethyl acetate/0.1% formic acid for 24 h at room temperature (2×). These extracts were concentrated *in vacuo*. The dried extracts were re-dissolved in methanol at 5 mg/ml and filtered for analysis on the Waters ACQUITY UPLC system using PDA UV and QDA mass detection using the following conditions: 0.5 ml/min, solvent A (0.1% formic acid in water); solvent B (0.1% formic acid in acetonitrile); 5% B (0–1.25 min), gradient to 25% B (1.25–1.5 min), gradient to 100% B (1.5–5.0 min), 100% B (5.0–7.5 min), then column equilibration 5% B (7.6–10.1 min). Peaks were identified using authentic standards. Cyclopiazonic acid (CPA) was purchased from Sigma-Aldrich (St. Louis, MO, United States). Aflavinine and aflatrem standards were kind gifts from Dr. James Gloer, University of Iowa, Iowa City, IA, United States.

### RNA Isolation, cDNA Synthesis, and Gene Expression Analysis

Total RNA was isolated from homogenized *A. flavus-*infected maize kernels using ‘Spectrum^TM^ Plant Total RNA kit’ (Sigma-Aldrich, St. Louis, MO, United States) and cDNA was synthesized using iScript^TM^ cDNA synthesis kit (Bio-Rad, Hercules, CA, United States) according to the manufacturer’s protocols. The ZR Fungal/Bacterial RNA MiniPrep^TM^ kit (Zymo Research, Irvine, CA, United States) was used for RNA extraction from *A. flavus* mycelial samples. Quantitative RT-PCR (qRT-PCR) was performed using SYBR green I chemistry and iCycler iQ5 Multicolor real-time PCR detection system (Bio-Rad). The thermocycler conditions included a pre-incubation at 95°C for 3 min, dye activation at 95°C for 10 s, primer annealing at 55°C for 30 s, elongation at 55°C for 50 s followed by a dissociation curve from 65 to 95°C for 30 min (with 0.5°C increments). The primers used for qRT-PCR are shown in Supplementary Table S2. Gene expression was normalized by ΔΔC_T_ analysis (Livak and Schmittgen, 2001) to *A. flavus β-tubulin* gene (AFLA_068620) or *Zea mays* ribosomal structural gene GRMZM2G024838 expression (Shu et al., 2015) utilizing the gene expression analysis software package of the Bio-Rad iQ5.

Determination of fungal load in the maize kernels infected with the WT and *Δspds A. flavus* CA14 strains were performed on 8 dpi samples. Fungal load was measured (using a method similar to that of Thakare et al. (2017) as relative expression of *A. flavus β-tubulin* gene (AFLA_068620) to the maize ribosomal structural gene GRMZM2G024838 (Shu et al., 2015).

### Statistical Analysis

Statistical significance between control and treatments were determined by Student’s *t*-test. Significant difference between control and treatment were analyzed at ^∗∗^*P* ≤ 0.05 and/or ^∗^*P* ≤ 0.10 as indicated in the legends of Figures and Table.

## Results

### Phenotypic Analyses of *A. flavus Δspds* Mutant and Genetic Complementation of the Mutant

**FIGURE 2:**
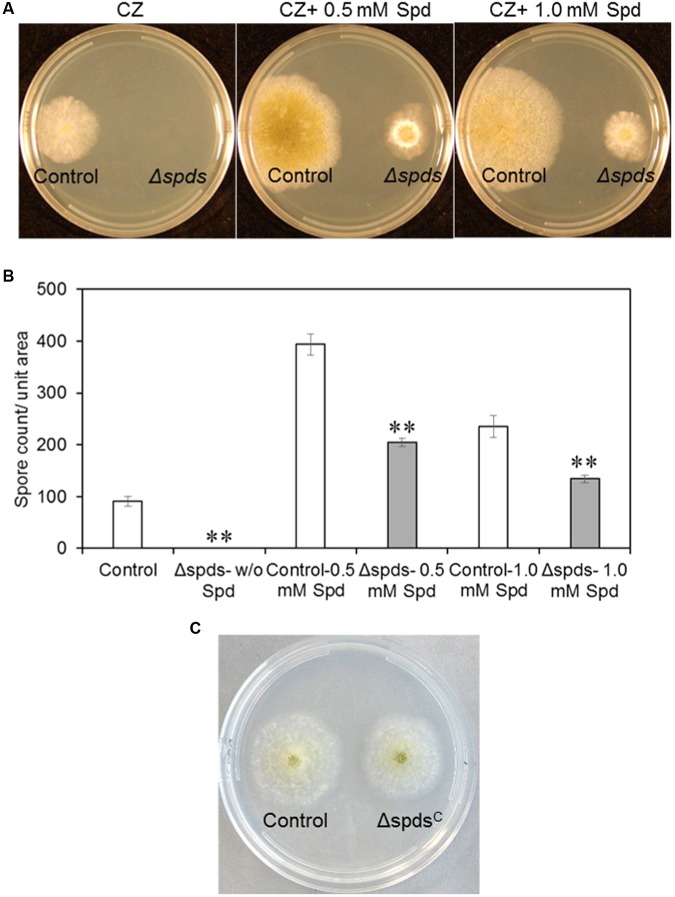
The effect of mutation in the *spds* gene (*Δspds*) on colony growth and sporulation. **(A)** Colony morphology of control (CA14 pyrG-1) and *Δspds* mutant *Aspergillus flavus* CA14 strains after 5 days of growth on Czapek’s (CZ) solid medium; **(B)** Comparison of conidial production between control and *Δspds* strains; and **(C)** Colony morphology of control and *Δspds* complemented strains. The cultures were grown under light for 5 days at 30°C. Data are Mean ± SE of three replicates (^∗∗^*P* ≤ 0.05 between control and treatment, Student’s *t*-test).

Disruption of the *spds* gene in the *A. flavus* CA14 strain was confirmed by PCR analysis, and a single representative knockout strain was selected for subsequent analyses (Supplementary Figure S1). Loss of *spds* gene expression in the selected knockout was confirmed by qRT-PCR (Supplementary Figure S1). Inactivation of *A. flavus spds* resulted in a total loss of growth and sporulation in the *Δspds* mutant as compared to the CA14 pyrG-1 control in the absence of exogenously supplied Spd in the CZ growth medium (**Figures 2A,B**). Addition of 0.5 mM Spd restored sporulation in the *Δspds* mutant but it was still <50% of that observed in the control grown in 0.5 mM Spd. An increase of Spd concentration in the CZ medium to 1.0 mM from 0.5 mM reduced sporulation by 34% in the *Δspds* mutant. A significant increase (334%) in spore production was observed in the control strain in response to 0.5 mM Spd compared to the *Δspds* mutant that was 49% less than the control at this concentration. An increase in Spd concentration to 1.0 mM had less promotional effect than 0.5 mM Spd on spore production in the control strain. Genetic complementation of the *Δspds* mutant (*Δspds^C^*) with a WT *A. flavus spds* gene restored host strain levels of sporulation and aflatoxin production without any exogenous supply of Spd (**Figure 2C** and Supplementary Figures S2, S3).

### Polyamine Content

**FIGURE 3:**
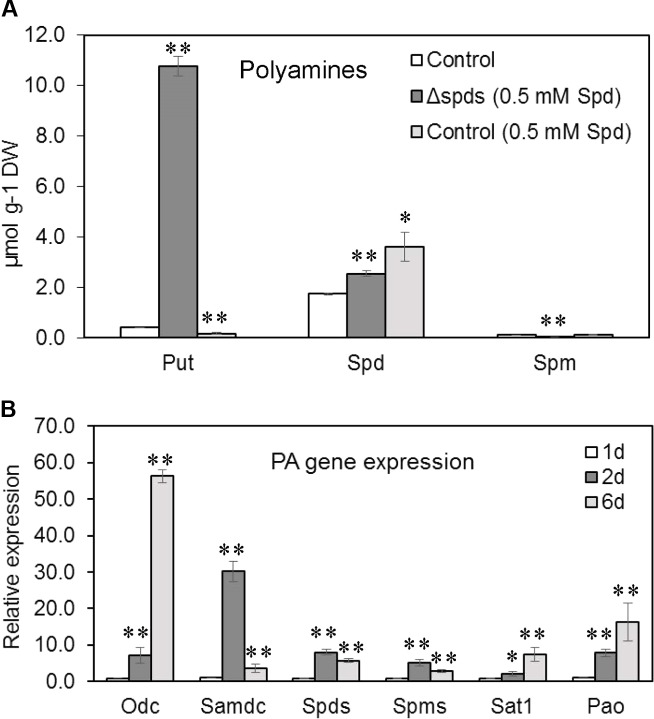
Polyamine contents and PA gene expression. **(A)** PA contents in the control (CA14 pyrG-1) and *Δspds A. flavus* CA14 strains grown in Czapek’s liquid medium (shake culture) with or without spermidine (Spd) for 8 days in the dark at 30°C; and **(B)** Developmental expression of PA genes (*odc*, *samdc*, *spds*, *spms*, *sat*1, *pao*) in *A. flavus* control strain (CA14 pyrG-1) grown in Czapek’s liquid medium (static culture) for 6 days in the dark at 30°C. Data are Mean ± SE of three replicates (^∗∗^*P* ≤ 0.05 and ^∗^*P* ≤ 0.10 between control and treatment or in comparison to day 1 in **B**, Student’s *t*-test).

Among the three different PAs analyzed (**Figure 3A**) in *A. flavus* mycelia, the concentration of Spd was highest (1.730 μM/mg DW), followed by Put (0.4 μM/mg DW) and Spm (0.1 μM/mg DW) in the control strain (CA14 pyrG-1) at 8 dpi when grown in CZ liquid medium. Addition of 0.5 mM Spd in the growth medium significantly decreased Put content by 134%, whereas cellular contents of Spd increased by 108% in the control. Whereas, in the *Δspds* mutant (supplemented with 0.5 mM Spd), cellular content of Put and Spd increased by 2619 and 52%, respectively, and Spm decreased by 2042% as compared to the control.

### Expression of Polyamine Biosynthetic Genes in *A. flavus* During Development

The expression of all PA biosynthetic genes (*odc*, *samdc*, *spds*, and *spms*) and PA back conversion genes (*pao*, *sat1*) studied increased by several fold from day 1 to day 2 during early developmental stages in the control strain (CA14 pyrG-1; **Figure 3B**). The change in expression of the *samdc* gene was the highest (31-fold) followed by *spds* (10-fold), *odc* (8-fold), *spms* and *pao* (6-fold), and *sat*1 (3-fold). At day 6, the expression of *samdc*, *spds*, and *spms* were lower than their corresponding expression values at day 2 while *odc*, *pao*, and *sat1* were up-regulated by 60-, 14-, and 9-fold, respectively, from their corresponding values at day 1.

### Amino Acids Analyses

The PA biosynthetic pathway is intimately associated with AA metabolism, therefore cellular AAs content was analyzed in the *Δspds* mutant along with the control (CA14 pyrG-1) *A. flavus* strains. Among the different PCA soluble AAs that were resolved by our HPLC method (**Table 1**), cellular content (% of the total soluble AAs) of Arg + Thr constituted the highest (30%) followed by Ala (16%), Orn (11%), and Lys (10%). Among the remaining AAs whose cellular content was between ≥10% were GABA (9%), Asp and Glu (7% each), Ser and Gly (3% each), and Pro (2%). The AAs that were<1% included Val, Ile, Leu, and Cys.

**Table 1:** Cellular content of amino acids (AAs) in the control (CA14 pyrG-1) and *Δspds Aspergillus flavus* CA14 strains grown in Czapek’s liquid medium (shake culture) with or without exogenous spermidine (Spd).

Treatment	Control (nmol/g dry wt)	*Δspds* (0.5 mM Spd) (nmol/g dry wt)	Control (0.5 mM Spd) (nmol/g dry wt)
Glu	4712.40 ± 428.94	3163.80 ± 219.81**	2987.40 ± 666.34
Orn	7421.20 ± 465.23	7159.47 ± 491.46	4428.33 ± 861.85*
Pro	1103.80 ± 73.81	1186.07 ± 16.98	840.33 ± 150.36
His	nd	1240.40 ± 114.74**	nd
Arg+Thr	19818.07 ± 945.68	13079.27 ± 4183.29	16423.47 ± 2522.16
GABA	5995.93 ± 257.44	5757.93 ± 88.34	5858.00 ± 1159.22
Ser	2125.80 ± 140.12	1651.00 ± 93.44*	1848.53 ± 270.65
Gly	1859.33 ± 150.76	1491.27 ± 56.75	1367.27 ± 239.29
Cys	85.40 ± 4.77	nd**	23.13 ± 23.13
Lys	6284.72 ± 253.33	5185.80 ± 329.61*	4241.67 ± 892.49
Ala	10516.67 ± 453.23	12194.40 ± 339.64**	6727.73 ± 1135.51*
Asp	4822.07 ± 386.00	2799.07 ± 304.30**	2858.40 ± 758.38
Val	596.40 ± 14.62	597.20 ± 18.45	604.20 ± 114.76
Ile	429.20 ± 4.72	424.33 ± 6.90	385.33 ± 81.91
Leu	425.90 ± 15.50	370.53 ± 20.16	463.13 ± 94.44

*The cultures were grown in the dark for 8 days at 30°C. Data are Mean ± SE of three replicates (^∗∗^P ≤ 0.05 and ^∗^P ≤ 0.10 between wild type (WT) and treatment, Student’s t-test); nd, not detected; Glu, glutamate; Orn, ornithine; Pro, proline; His, histidine; Arg+Thr, arginine+threonine; GABA, γ-aminobutyric acid; Ser, serine; Gly, glycine; Cys, cysteine; Lys, lysine; Ala, alanine; Asp, aspartate; Val, valine; Ile, isoleucine; Leu, leucine.*

In general, the AAs whose cellular content (nmol/g DW basis) decreased significantly in the *Δspds* mutant (vs. control) were Asp (42%), Glu (33%), Ser (22%), Lys (17%), and Cys (not detected in the *Δspds* mutant). The AAs that increased significantly in the *Δspds* mutant with Spd (vs. control) were Ala (16%) and His (not detected in any other treatments). The AAs that were changed in the control with 0.5 mM Spd vs. control (no Spd) were, Ala (36% decrease) and Orn (40% decrease).

### Effect of Exogenous Supply of Spd and Spm on Sclerotia Production

**FIGURE 4:**
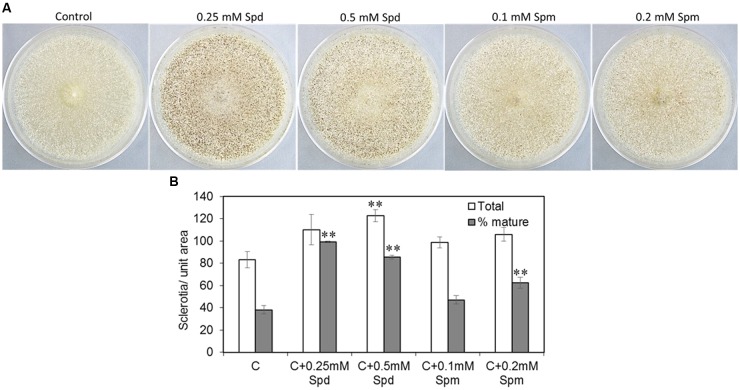
Spermidine affects sclerotial formation. **(A)** Sclerotial morphology of *A. flavus* AF70 wild type (WT) strain grown on solid Czapek’s (CZ) medium supplemented with different concentrations of Spd and Spm along with control (no PAs); and **(B)** Mature (melanized) vs. total sclerotia counts for the AF70 strain grown on solid CZ medium supplemented with Spd and Spm along with control (no PAs). The cultures were grown in the dark for 14 days at 30°C. Data are Mean ± SE of four replicates (^∗∗^*P* ≤ 0.05 between control and treatment, based on Student’s *t*-test).

To investigate if PAs promoted sclerotial production, CZ medium supplemented with PAs was used to inoculate the *A. flavus* AF70 strain (a high sclerotia-producing strain). Exogenous supply of PAs significantly increased the number of sclerotia and the percentage of mature (melanised) sclerotia when the AF70 strain was grown on CZ medium supplemented with Spd or Spm (**Figures 4A,B**). Supplementing CZ medium with 0.25 or 0.5 mM of Spd increased the total number of sclerotia by 32 and 47%, respectively, as compared to the non-supplemented control (**Figure 4B**). The effect of Spd was more pronounced on the production of mature sclerotia resulting in a 61 and 57% increase with 0.25 and 0.5 mM Spd, respectively (vs. non-supplemented control). Supplementation of CZ medium with Spm significantly increased the number of mature sclerotia by 24% at 0.2 mM. No significant changes in sclerotia production were observed at any other concentration of Spm used.

### Effect of Exogenous Supply of Spd and Spm on Secondary Metabolites Production, and Associated Gene Expression in *A. flavus*

**FIGURE 5:**
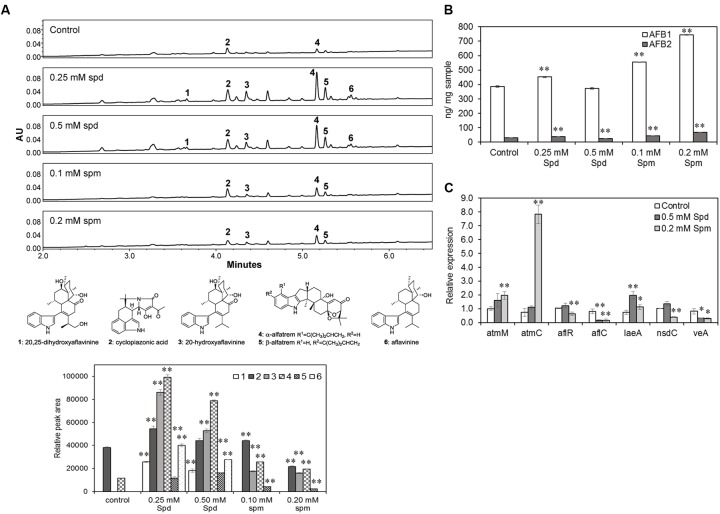
The effects of Spermidine and Spermine (Spm) on secondary metabolite (SM) production and related gene expression. **(A)** Production of indole diterpene SMs (chromatograms at λ = 254 nm) in *A. flavus* AF70 WT strain grown on solid Czapek’s medium (with ammonium sulfate) supplemented with different concentrations of Spd and spermine along with control (no PAs) grown for 14 days in the dark at 30°C. Aflatoxins were not detected in any of these samples as Czapek’s medium is not conducive for aflatoxin production; **(B)** Production of aflatoxins in *A. flavus* AF70 WT strain grown on solid A&M medium supplemented with different concentrations of Spd and spermine along with control (no PAs) grown for 7 days in the dark at 30°C; **(C)** Effect of exogenous supply of Spd and Spm on the expression of secondary metabolite related genes, *atmM*, *atmC*, *aflR*, *aflC*, *cpaA*, and global regulatory genes, *laeA*, *nsdC*, and *veA* in *A. flavus* AF70 WT strain. The samples were grown in CZ liquid medium (static culture) for 5 days in the dark at 30°C. Data are Mean ± SE of three replicates (^∗∗^*P* ≤ 0.05 and ^∗^*P* ≤ 0.10 between control and treatment, Student’s *t*-test).

*Aspergillus flavus* 70, a producer of high levels of aflatoxins and sclerotia, was used to study the effects of PAs on SM production. UPLC-MS analysis of AF70 extracts grown on CZ medium (with ammonium sulfate) in the presence of Spd showed an overall significant increase in the production of SMs compared to the control (untreated) or Spm treated samples. Indole diterpenes, particularly aflavinines (1, 3, 6; **Figure 5A**) and aflatrems (4, 5; **Figure 5A**), and CPA (2; **Figure 5A**) were significantly increased when Spd was added to the culture medium. Spermine also significantly increased the production of aflavinines, aflatrems, and CPA that varied with the Spm concentration. The overall effect of Spd on the production of aflavinines and aflatrems was higher than Spm. CZ medium is not conducive for the production of aflatoxins, therefore, A&M medium, which contains ammonium sulfate and supports aflatoxin biosynthesis was used to study the effects of PAs on aflatoxin production. Spermine (as compared to Spd) had a greater impact on the production of aflatoxins. At 0.1 and 0.2 mM Spm there was a significant increase in AFB1 content of 44 and 92%, respectively (vs. control; **Figure 5B**), and AFB2 content by 52 and 135%, respectively (vs. control). Spd on the other hand, at 0.25 mM increased AFB1 and AFB2 content by 17 and 28%, respectively, and decreased AFB2 by 14% at without any significant change in AFB1 at 0.5 mM concentration (vs. control; **Figure 5B**).

As PAs in general, significantly increased SM production, we wanted to determine if this was due to up-regulation of SM pathway-specific and global regulatory genes. Expression of biosynthetic genes associated with production of aflavinine and aflatrem (*atmC;* AFLA_096390 and *atmM*; AFLA_096400), CPA (*cpaA*; AFLA_139490) as well as the global regulators of secondary metabolism, *nsdC* (AFLA_131330)*, laeA* (AFLA_033290), and *veA* (AFLA_066460) was determined following 5 days growth of AF70 in CZ broth supplemented with Spd or Spm. The expression of *atmM* and *atmC* were both up-regulated by Spm (**Figure 5C**), especially *atmC* up-regulated (10.7-fold) by 0.2 mM Spm compared to the control or Spd treated sample. Expression of *laeA* was increased by 2.6-fold in 0.5 mM Spd with a small but significant increase in response to Spm. Spermine (0.2 mM) down-regulated the expression of *veA* and *nsdC* by 2- to 3-fold whereas a small increase in the expression of *nsdC* and decrease in *veA* expression was observed by 0.5 mM Spd. No significant change in the expression of *cpaA* was observed in response to Spd or Spm treatments at 5 days of growth.

### Analysis of Fungal Growth and Aflatoxin Production in Infected Maize Kernels

**FIGURE 6:**
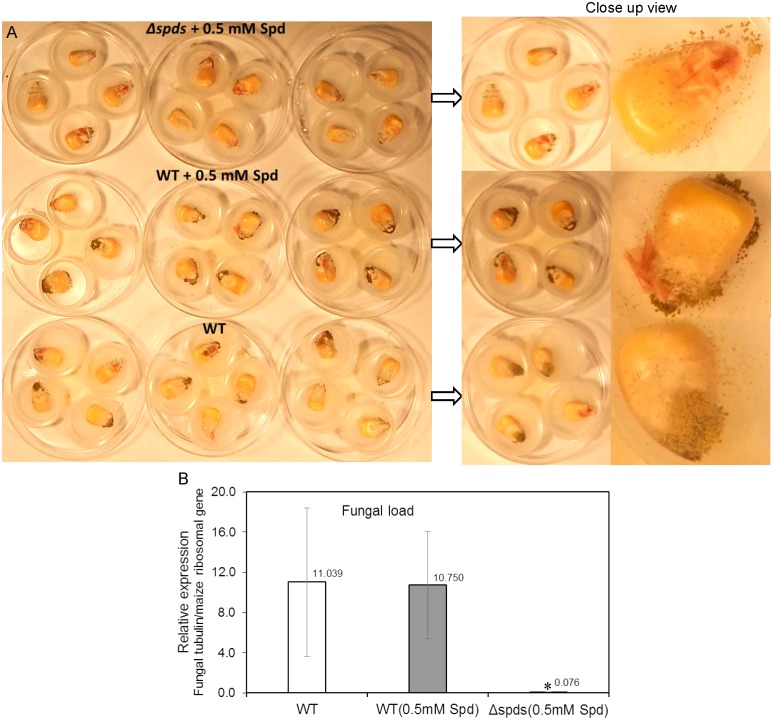
The CA14 *Δspds* mutant showing reduced pathogenicity during maize-*A. flavus* interaction. **(A)** Maize kernels at 8 days post-inoculation (dpi) were infected with either the CA14 WT or the *Δspds* mutant (post-growth on CZ solid medium, which may or may not have been supplemented with Spd, for 1 week prior to the kernel inoculation); **(B)** Estimation of fungal load in maize kernels at 8 dpi, infected with WT and *Δspds* CA14 strains. Fungal load was expressed as relative expression of the *β-tubulin* gene (AFLA_068620) to the maize ribosomal structural gene (used as housekeeping) GRMZM2G024838 (Shu et al., 2015). Data are Mean ± SE of 3–4 replicates, each replicate consists of 4–5 seeds; (^∗^*P* ≤ 0.10 between WT and treatment, Student’s *t*-test).

A maize seed infection assay using *A. flavus* WT CA14 and *Δspds* was performed to investigate if s*pds* plays a role in pathogenicity and aflatoxin production during seed colonization. The *Δspds* mutant (a Spd auxotroph) was grown on CZ medium supplemented with ammonium sulfate and 0.5 mM Spd for 8 days along with WT grown on CZ medium (with or without 0.5 mM Spd) for the same time period prior to the harvest of spores for maize seed inoculation. In general, seeds infected with the WT strain (with or without Spd treatment) highly sporulated on the seed surface as opposed to the *Δspds* mutant strain that produced less spores (**Figure 6A**). Estimation of fungal load within the seeds showed a 140 to 144-fold higher growth in the WT *A. flavus* infected seeds as compared to *Δspds* mutant infected seeds (**Figure 6B**).

**FIGURE 7:**
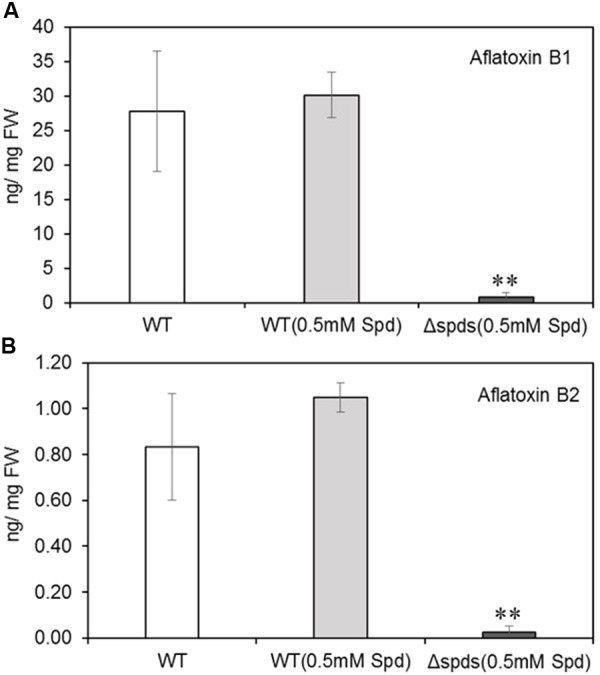
Aflatoxin contents in maize kernels infected with WT and *Δspds A. flavus* CA14 strains. **(A)** Aflatoxin B1; and **(B)** Aflatoxin B2. Data are Mean ± SE of four replicates, each replicate consists of 4–5 seeds (^∗∗^*P* ≤ 0.05 between WT and treatment, Student’s *t*-test).

Aflatoxin analysis of maize seeds infected with WT *A. flavus* (with or without Spd) produced significantly higher amounts of aflatoxins (18–30 ng/mg FW AFB1 and 0.8–1.0 ng/mg FW AFB2; **Figures 7A,B**) than the *Δspds* infected seeds (7.74 ng/mg FW AFB1 and 0.26 ng/mg FW AFB2, respectively; **Figures 7A,B**).

### Expression Analyses of Aflatoxin and Polyamine Metabolism and Transport Genes in *A. flavus* and Maize

**FIGURE 8:**
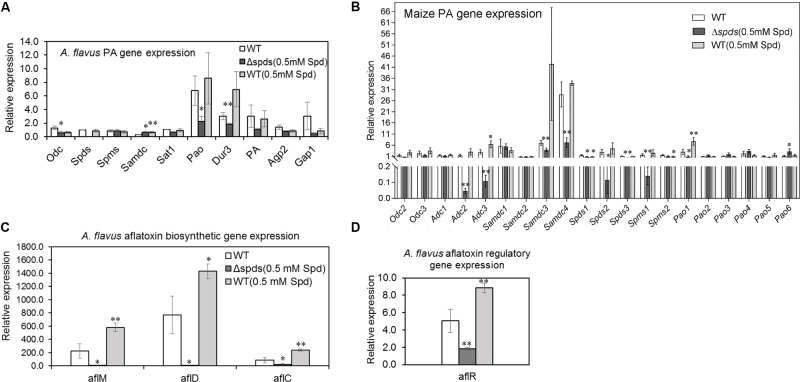
*Aspergillus flavus* CA14 *Δspds* mutant shows changes in PA and aflatoxin gene expression during maize-*A. flavus* interaction. Maize kernels were infected with WT and *Δspds* strains for 8 days, and gene expression was analyzed in the infected maize kernels at 8 days post-inoculation (dpi). **(A)** Expression of *A. flavus* PA biosynthetic genes (*odc*, *spds*, *spms*, *sat*1) and putative plasma membrane localized PA uptake transporters (*dur*3, *PA*, *agp*2, *gap*1) during host-pathogen interaction; **(B)** Expression of maize PA metabolism genes (*Odc*, *Adc*, *Samdc*, *Spds*, *Spms*, *Pao*) in the kernels during *A. flavus* infection; **(C)** Expression analyses of *A. flavus* aflatoxin biosynthetic genes (*aflM*, *aflD*, *aflC*); and **(D)** Expression of aflatoxin regulatory gene *aflR* in maize kernels infected with WT and *Δspds* CA14 strains. Data are Mean ± SE of 3–4 replicates, each replicate consists of 4–5 seeds (^∗∗^*P* ≤ 0.05 and ^∗^*P* ≤ 0.10 between WT and treatment, Student’s *t*-test).

During maize seed infection, the expression of putative PA transporter and *pao* genes was generally higher in the WT *A. flavus* strain (control) in comparison to the expression of other PA biosynthetic genes (**Figure 8A**). Among the different putative PA uptake transporters (*dur3*, AFLA_029660; *pa*, AFLA_024200; *gap1*, AFLA_073560; and *agp2*, AFLA_113740) that were identified as yeast orthologs in *A. flavus*, expression of *dur3* was up-regulated (statistically significant) by 1.7-fold in the WT vs. *Δspds* mutant strain. Among the PA biosynthetic genes, expression of *odc* (AFLA_011800) and *pao* (AFLA_118340) was down-regulated by 2- and 3-fold, respectively, in the *Δspds* mutant compared to WT. Expression of *samdc* (AFLA_006490) gene was 2-fold higher in the *Δspds* mutant and WT (0.5 mM Spd) vs. WT.

Different genes of the maize PA biosynthetic pathway as well as PA catabolism were analyzed to see if expression of genes associated with PA metabolism in the seeds respond differently when subjected to WT (virulent) vs. *Δspds* mutant (less virulent) strains (**Figure 8B**). It is known that maize has multiple copies each of PA metabolism gene (Supplementary Table S2). Overall, the expression of *ZmSamdc* genes were highest followed by *ZmAdc* genes as compared to the other PA genes. Expression of *ZmOdc* genes, *ZmOdc2* (XM_008672579) and *ZmOdc3* (XM_008654778) did not vary much between samples and no expression of *ZmOdc1* (NM_001148682) was detected in any of the samples. Among the different *ZmAdc* genes studied here, expression of both *ZmAdc2* (NM_001138726) and *ZmAdc3* (XM_023300699) genes were up-regulated in the seeds infected with the WT strain only (vs. Δ*spds* mutant) with no expression of the *ZmAdc4* (XM_008671538) gene in any of the samples. Expression of *ZmSamdc3* and *ZmSamdc4* were significantly high (>10-fold higher than other genes) and were up-regulated in the seeds infected with the WT strain (vs. Δ*spds* mutant strain). Expression of *ZmSpds3* (NM_001155814) and *ZmSpms1* (NM_001112372) were higher in the seeds infected with the WT strain vs. Δ*spds* mutant strain. Among the six *ZmPao* genes, expression of *ZmPao1* (NM_001111636) was highest and was slightly up-regulated in the seeds infected with the WT strain as compared to the Δ*spds* mutant strain. No significant changes were observed in the expression of other *ZmPao* genes.

Among the aflatoxin biosynthetic genes in *A. flavus*, expression of *aflM* (AFLA_139300), *aflD* (AFLA_139390), *aflC* (AFLA_139410), and *aflR* (AFLA_139360) was significantly up-regulated by 110, 350, 100, and 3.5-fold, respectively, in the WT as compared to the *Δspds* mutant strain during seed infection (**Figures 8C,D**). The WT strain (0.5 mM Spd) had 1.4 to 2.8-fold higher expression of the aflatoxin biosynthetic genes in comparison to the WT strain grown on CZ agar medium without Spd (prior to the inoculation) during seed infection (**Figure 8C**).

## Discussion

### Ubiquitous Role of PAs in Fungal Growth, Development and Pathogenicity

Polyamines are ubiquitous in living organisms and are involved in regulating several cellular processes including growth, differentiation, stress response, and pathogenesis (reviewed in Valdés-Santiago et al., 2012; Minocha et al., 2014; Miller-Fleming et al., 2015). Thus, the PA biosynthetic pathway has often been the target of novel strategies to control diseases associated with fungal and other microbial pathogens (Amano et al., 2015; Burger et al., 2015; Estiarte et al., 2017; Mounce et al., 2017). During the pathogenic interaction between the fungus *Colletotrichum truncatum* and soybean seeds, application of L-α-difluoromethylornithine (DFMO) and difluoromethylarginine (DFMA), inhibitors of Odc and Adc, respectively, reduced intracellular PAs in the fungi and significantly impacted fungal growth (Gamarnik et al., 1994). Exogenous supply of Put or Spd restored fungal growth, suggesting that intracellular contents of Spd might be critical for growth and pathogenesis. The current study, using a gene knock out approach, directly demonstrates that Spd is critical in *A. flavus* growth, development, pathogenesis, and the production of aflatoxins *in vitro*; especially during the maize-*A. flavus* interaction. The *Δspds* mutant was an auxotroph for Spd that required an exogenous supply of Spd to partially restore WT levels of growth and sporulation. The inability of Spd supplementation to fully reverse the *Δspds* mutation suggested a differential regulation of Spd uptake and distribution in the fungus as compared to its endogenous biosynthesis. Nevertheless, exogenous supply of Spd significantly increased sporulation in the control strain suggesting regulation of sporulation by Spd (**Figure 2B**). The observation of a 6- to 31-fold higher expression from day 1 to day 2 of *A. flavus* PA biosynthetic genes (**Figure 3B**) indicates that there is a high demand for PAs to support the early stages of fungal growth and development. The inability of the *Δspds* mutant strain to produce Spd resulted in a significant reduction in fungal growth and aflatoxin production during maize seed infection (**Figures 6**, **7**). The results presented here are in line with earlier reports on the role PAs in fungal development and secondary metabolism. In *Fusarium graminearum* application of DFMO significantly reduced fungal growth (∼70%) and the production of DON by ∼53-fold, under *in vitro* conditions (Crespo-Sempere et al., 2015). A similar reduction in growth, sporulation, and SM production upon inhibition of Odc or *spds* knock out were also reported in *A. nidulans* and *A. parasiticus* (Khurana et al., 1996; Guzmán-de-Peña and Ruiz-Herrerai, 1997; Guzmán-de-Peña et al., 1998; Jin et al., 2002; Khatri and Rajam, 2007). The data presented here demonstrate the role of Spd in *A. flavus* growth and development in relation to the expression of PA biosynthetic genes and cellular PA content *in vitro* and during maize seed infection.

### Inactivation of Spds Alters Nitrogen Metabolism in the *Δspds* Mutant

Nitrogen metabolism plays a central role during normal fungal development as well as under stress conditions (Krappmann and Braus, 2005; Valdés-Santiago and Ruiz-Herrera, 2013). PAs can alter overall N metabolism as the PA biosynthetic pathway is intricately associated with AA biosynthesis (Mohapatra et al., 2010; Beckmann et al., 2013; Majumdar et al., 2013, 2016; Dalton et al., 2016; Wuddineh et al., 2018). Inhibition of Spd biosynthesis in *A. flavus* resulted in a significantly higher accumulation of Put (∼2619%) than the control (**Figure 3A**). Putrescine over-production is often accompanied by higher Put catabolism and rapid (within hours) turn-over of Put in plants (Bhatnagar et al., 2002; Shao et al., 2014). This might not be the case in *A. flavus* as a significant accumulation of Put was observed even after 8 days of culture. This could possibly be due to a greater half-life of Put in the fungus and previous observations that Put can be stored at a very high concentration (under certain conditions) in cells, where it might serve as a source of N during low N availability (Middlehoven et al., 1986). The accumulation of Put in the *Δspds* mutant of *A. flavus* could be a topic for future studies where radio-labeled PA substrates can be used to study Put turn-over rates and the possible back-conversion of Spd to Put by Sat and Pao enzymes. Other than PAs, intracellular AA content also has a substantial impact on overall N metabolism in fungi and AAs play an indispensable role in fungal development and pathogenesis. Thus, inhibitors of AA metabolism have been used as antifungal agents (Jastrzebowska and Gabriel, 2015). In the current study, supply of exogenous Spd to the *Δspds* mutant revealed that although *A. flavus* resumed growth and sporulation *in vitro* (albeit lower than the control), intracellular AA content in the mutant was still not on par with the WT strain (**Table 1**). Alterations in cellular PAs due to inactivation of PA biosynthetic genes (*spds*, *odc*) in *A. nidulans* were reported in earlier studies (Jin et al., 2002; Khatri and Rajam, 2007), but the current data show that alterations in Spd levels in *A. flavus* goes beyond the PA biosynthetic pathway and affects biosynthesis of AAs. Several of the AAs (such as Glu, Ser, Thr) that were decreased in the *Δspds* mutant (**Table 1**) are reported to be critical in fungal pathogenesis (Olivieri et al., 2002; de Sain and Rep, 2015; Muszewska et al., 2017; Zhou et al., 2017). Whether the reduction in pathogenicity in the *Δspds* mutant during maize seed infection is due to the decrease in Spd or possible alteration in relative AA content or both, will require further investigation.

### Polyamines Modulate Secondary Metabolite (SM) Production

The PA-hypusine node is critical for cell survival and significantly affects pathogenicity in fungi and other organisms (reviewed in Park et al., 2010; Wolff and Park, 2015). The conversion of a specific Lys residue in the eIF5A to a rare AA hypusine (post-translational modification) and subsequent activation of eIF5A is absolutely required for cell proliferation. Spd in this regard serves as the only donor of a 4-aminobutyl moiety to the Lys residue of eIF5A carried out by the enzyme deoxyhypusine synthase. The overall effect of Spd depletion on growth and SM production in the *Δspds* mutant may be due to a combination of reduced activation of eIF5A leading to reduced fungal growth and direct regulation of SM biosynthesis by Spd (Martinez-Rocha et al., 2016). Supplementation of CZ minimal medium with Spd and to some extent Spm significantly increased the formation of sclerotia and production of aflavinines and aflatrems in AF70 (**Figures 4**, **5A**). A significant increase in intracellular PA content has been shown to be necessary for sclerotial development in *Sclerotinia sclerotiorum* (Pieckenstain et al., 2001). The results presented here further demonstrate the direct role of PAs in the production of sclerotia and associated SMs in *A. flavus*. Besides sclerotial metabolites, production of aflatoxins was also increased by supplementation of the medium with Spd and even greater increases were observed at both concentrations of Spm tested (**Figure 5B**). The effect of *spds* inactivation (*Δspds* mutant) on aflatoxin production was more pronounced during infection of maize seeds where aflatoxin levels were significantly reduced compared to those measured in WT fungal infections (**Figure 7**). The results obtained from *in vitro* studies suggest a differential mode of regulation of SM-related gene expression by Spd vs. Spm. Significant up-regulation of the *laeA* global regulatory gene by Spd supplementation in the growth medium (**Figure 5C**) and subsequent increase in SM production (**Figure 5A**), might indicate a possible mode of action of Spd to increase SM production. This observation is in line with the earlier work in *Penicillium chrysogenum* (*P. chrysogenum*), where exogenous supply of Spd or 1,3-diaminopropane (1,3-DAP; a catabolic product of Spd) to the *laeA* knockdown mutant (produces very low amounts of benzyl-penicillin) significantly increased the expression of penicillin biosynthetic genes and completely restored the production of benzyl-penicillin in the *laeA* mutant to WT levels (Martin et al., 2012). An observed increase in the levels of other uncharacterized *A. flavus* SMs upon supplementation of media with Spd also points to PAs having the capacity to potentially activate silent metabolic gene clusters (data not shown). Biochemical characterization of these SMs could be of interest for future studies. Expression of *veA* and *nsdC*, global regulators of development and secondary metabolism in *A. flavus* (Cary et al., 2007, 2012; Duran et al., 2007), were also studied with respect to their regulation by PAs. Expression of *nsdC* was slightly increased by Spd whereas both Spd and Spm slightly repressed the expression of *veA* (**Figure 5C**). Future RNA-seq studies using the *A. flavus Δspds* mutant will help in understanding the global regulation of gene expression by Spd as it relates to growth, development, pathogenicity, and the production of SMs.

### PA Uptake and Biosynthesis Plays a Key Role in Fungal Pathogenesis

Polyamine transport in fungi plays a major role in fungal growth during host-pathogen interactions (reviewed in Valdés-Santiago et al., 2012; Miller-Fleming et al., 2015; Estiarte et al., 2017). In the model fungus *Saccharomyces cerevisiae*, among several plasma membrane localized transporters, Dur3, Sam3, Agp2, and Gap1 are reported to be involved in PA uptake from the external environment with Dur3 and Sam3 being the major importers of extracellular PAs (Aouida et al., 2005, 2013; Uemura et al., 2005, 2007). In *A. nidulans*, Put uptake is 2- to 3-fold more rapid than the uptake of Spd (Spathas et al., 1982) and uptake of Spd was inhibited by increased concentrations of Put and Spm. However, Put uptake was not affected by the presence of Spd or Spm. Expression analyses of PA genes and transporters in *A. flavus* during maize seed infection showed a significant increase in the expression of putative PA uptake (Put?) transporter *dur3* (AFLA_029660) in the WT compared to the *Δspds* mutant indicating an important role for PA uptake by the fungus to achieve successful pathogenesis (**Figure 8A**). The fact that Put content increases several-fold in plants during fungal infection and uptake of Put is more efficient than other PAs (Spathas et al., 1982; Wojtasik et al., 2015), it is likely that Put is the predominant PA that the fungus might uptake from the plant host. Application of biochemical inhibitors of putative plasma membrane localized PA uptake transporters and significant reduction in fungal infection and DON production (reduced 124-fold) were reported during infection of wheat spikelets by *F. graminearum* (Crespo-Sempere et al., 2015). Similar results on inhibition of PA transport and reduction in fungal pathogenicity *in vitro* and *in vivo* were reported for *Alternaria alternata* infection of tomato (Estiarte et al., 2017). Among the expression of different PA metabolism genes that were studied during the maize-*A. flavus* interaction, the expression of *ZmSamdc* genes was the highest followed by *ZmAdc* genes, that significantly increased in the maize kernels following inoculation with the WT *A. flavus* strain compared to the *Δspds* mutant (**Figure 8B**). This suggested differential responses in maize Put biosynthesis and production of higher PAs with respect to virulent vs. less virulent strains of the same pathogen. Similar differential induction of Put biosynthesis by *Odc* (higher against virulent strain) in flax seedlings was observed when they were exposed to virulent and avirulent strains of *Fusarium culmorum* and *Fusarium oxysporum*, respectively (Wojtasik et al., 2015). Significant increase in the expression of maize *Samdc* and *Adc* genes were also reported in leaves infected by the fungal pathogen *Ustilago maydis* (Rodríguez-Kessler et al., 2008). Overall the results presented here suggest that up-regulation of PA uptake transporters in *A. flavus* during infection of maize leads to PA uptake from the host though this cannot fully compensate for Spd depletion in the *Δspds* mutant.

## Conclusion

Polyamine metabolism plays a significant role in host defense and also in maintaining successful pathogenesis in fungi (and in other pathogens) during the host-pathogen interaction. The results presented here demonstrate the role of Spd in *A. flavus* growth, development, and SM production both *in vitro* and *in vivo*. Abrogation of intracellular Spd biosynthesis in *A. flavus* negatively affected fungal growth, expression of PA biosynthetic genes, and aflatoxin biosynthesis during maize seed infection. Increase in overall SM production in *A. flavus* by an exogenous supply of Spd (*in vitro*) also supports the role of Spd in SM production. Significant up-regulation of *A. flavus* plasma membrane-localized PA uptake transporters during maize seed infection suggests that dual targeting of PA uptake transporters and *spds* through an RNAi- based approach or application of biochemical inhibitors, might be an effective strategy to control *A. flavus* colonization and aflatoxin production in maize and other susceptible food crops.

## Author Contributions

RMa, JC, SM, and KR: conceived and designed the experiments. RMa, BM, and CS: performed the experiments. RMa, ML, RMi, SM, and CC-W: analyzed the data. RMa, ML, and RMi: wrote the paper. JC, RMi, SM, and KR: edited the draft manuscript. All authors reviewed and approved the final manuscript.

## Conflict of Interest Statement

The authors declare that the research was conducted in the absence of any commercial or financial relationships that could be construed as a potential conflict of interest.

## Abbreviations

AA: amino acid
Adc: arginine decarboxylase
Ala: alanine
Arg: arginine
Asp: aspartate
Cys: cysteine
Dao: diamine oxidase
GABA: γ-aminobutyric acid
Glu: glutamate
Gly: glycine
His: histidine
Ile: isoleucine
Leu: leucine
Lys: lysine
Odc: ornithine decarboxylase
Orn: ornithine
PA: polyamine
Pao: polyamine oxidase
PCA: perchloric acid
Pro: proline
Put: putrescine
Samdc: S-adenosylmethionine decarboxylase
Ser: serine
SM: secondary metabolite
Spd: spermidine
Spds: spermidine synthase
Spm: spermine
Spms: spermine synthase
Thr: threonine
Trp: tryptophan
Val: valine
WT: wild-type
