# Supplementary material for: The Aspergillus flavus Spermidine Synthase (spds) Gene, Is Required for Normal Development, Aflatoxin Production, and Pathogenesis During Infection of Maize Kernels
Source: Front Plant Sci. 2018 Mar 20;9:317. doi: 10.3389/fpls.2018.00317 (PMC5870473; doi:10.3389/fpls.2018.00317)
Supplement: Supplementary file 1 [file Data_Sheet_1.DOCX]

**Table S1.** Oligonucleotide primers used for the construction of *A. flavus* CA14 *Δspds* gene knockout and complementation PCR products (lower case alphabets denote overlap sequences).

| **Primer name** | **Sequence (5’-3’)** | **Purpose** |
| --- | --- | --- |
| AFLA_017920 (*Spds*)_5F | TGCTTTGCGCGGAATAATCG | knockout |
| AFLA_017920 (*Spds*)_5R | gcaataagcccaaccctatcggcGAAAGCGAAGAAGATGCGCG | knockout |
| AFLA_017920 (*Spds*)_3F | tttgtaccggagtgtctgaaggTTCTTCCCAACTTTGCCCGT | knockout |
| AFLA_017920 (*Spds*)_3R | CCCCAGCTTGAGAACTCCAG | knockout |
| AFLA_017920 (*Spds*)_nest-F | ATGCTGATCTGCCAGGATCG | knockout |
| AFLA_017920 (*Spds*)_nest-R | GTCTTCGATGCCCAGGACAA | knockout |
| *pyrG*_F | GCCGATAGGGTTGGGCTTATTGC | knockout |
| *pyrG*_R | CTTCAGACACTCCGGTACAAATTCTC | knockout |
| 017920prom_F2 | TTTTGGTGGCGTTTGTTGCA | complementation |
| 017920term_R | tatagaagcacttaccttcgcatGAGAAGATCTGGAGGGCAGC | complementation |
| *pyrG*_F1 | ATGCGAAGGTAAGTGCTTCT | complementation |
| *pyrG*_R1 | TGAAACATGACCCTTGACTCTGA | complementation |
| 017920prom_nest-F | GGTCACTAGTGTTGAAGCGC | complementation |
| *pyrG*_nest-R | GTCCATATCTCGAGGCAGGC | complementation |

**Table S2.** Oligonucleotide primers used for the qRT-PCR work.

| **Primer name** | **Sequence (5’-3’)** |
| --- | --- |
| AFLA_068620 (*β-tubulin*)-qF | AGAGCAAGAACCAGACCTAC |
| AFLA_068620 (*β-tubulin*)-qR | GACGGAACATAGCAGTGAAC |
| AFLA_011800 (*Odc*)-qF | ATGCGAATTCTGCACGAAGC |
| AFLA_011800 (*Odc*)-qR | TCTCGTGTCGTTTCCAGTCG |
| AFLA_017920 (*Spds*)-qF | TTCGTTCGCACTTCTCCCA |
| AFLA_017920 (*Spds*)-qR | CGGAGAACCAGCCATCCT |
| AFLA_091840 (*Spms*)-qF | CTCACCGAGTAGACGCAGAC |
| AFLA_091840 (*Spms*)-qR | GTGATGAGAGCAGCAGGTGT |
| AFLA_006490 (*Samdc*)-qF | TGAGGTGACGCAGGATCTTG |
| AFLA_006490 (*Samdc*)-qR | GGCAAACCAGACCTCCAGAA |
| AFLA_014540 (*Sat1*)-qF | TCCGCGAACTAGCCGATTAC |
| AFLA_014540 (*Sat1*)-qR | GCGGTATATACGGAGCCTCG |
| AFLA_118340 (*Pao*)-qF | GGCCAGGACAAAGATGGGAA |
| AFLA_118340 (*Pao*)-qR | AGGACCTCCTGGATTTCCGA |
| AFLA_029660 (*Dur3*)-qF | CATCGCATGGCTTGTAACCG |
| AFLA_029660 (*Dur3*)-qR | ACGTTACCAGCGAGCATAGG |
| AFLA_024200 (*PA*)-qF | GCCGTGGGCCCAATCTATG |
| AFLA_024200 (*PA*)-qR | TGGCGGAAATATGGTAGACCC |
| AFLA_113740 (*Agp2*)-qF | GGAGCGATGTGGTACCAACA |
| AFLA_113740 (*Agp2*)-qR | TCCCAGAGCAAGCCAAAACT |
| AFLA_073560 (*Gap1*)-qF | CGGTGAGGCTGAGTTTGTCT |
| AFLA_073560 (*Gap1*)-qR | AGTTCAGGACAATGCCGAGG |
| AFLA_139410 (*aflC*; *pksA*)-qF | CGCCACCTATTTTGCCGATG |
| AFLA_139410 (*aflC*; *pksA*)-qR | GTACTCAGACACAGACCGGC |
| AFLA_139390 (*aflD*; *nor-1*)-qF | CAGCACCATCACCAACATGC |
| AFLA_139390 (*aflD*; *nor-1*)-qR | CTGCACATGTCCTGGATCGA |
| AFLA_139360 (*aflR*)-qF | CTCAAGGTGCTGGCATGGTA |
| AFLA_139360 (*aflR*)-qR | CAGCTGCCACTGTTGGTTTC |
| AFLA_139300 (*aflM*; *ver-1*)-qF | CGCCACCTATTTTGCCGATG |
| AFLA_139300 (*aflM*; *ver-1*)-qR | GTACTCAGACACAGACCGGC |
| AFLA_096400 (atmM)-qF | GGTGTCGAAGCGGGAAAAGA |
| AFLA_096400 (atmM)-qR | CTCCGACCTTTAAGCCTGGA |
| AFLA_096390 (atmC)-qF | TCCTAGAGATCATGGACGGAA |
| AFLA_096390 (atmC)-qR | CGATAGTCACTGCCGTCACT |
| AFLA_033290 (*laeA*)-qF | TCGGATGGCCTAATGTACGC |
| AFLA_033290 (*laeA*)-qR | AGCAAGGTCAACCCCAACAA |
| AFLA_131330 (*nsdC*)-qF | CTTCATCGCGCTCACTCTCC |
| AFLA_131330 (*nsdC*)-qR | GGTTGCTAGAATGGCTGTGG |
| AFLA_066460 (*veA*)-qF | CGCCAAGAAGTTCCCAGGAT |
| AFLA_066460 (*veA*)-qR | TTCTCTCCCCGACGTCTCAT |
| *Zm*_NM_001148682 (*Odc1*)-qF | TTCCGCATTGACAACGTCCT |
| *Zm*_NM_001148682 (*Odc1*)-qR | TTGCACTTGACGGCGTAGTA |
| *Zm*_XM_008672579 (*Odc2*)-qF | CATGCATGCTGCAGAGCAAA |
| *Zm*_XM_008672579 (*Odc2*)-qR | AACCCAAGTCCGAAGGCAAT |
| *Zm*_XM_008654778 (*Odc3*)-qF | CCCCGGCGATAAAACCTACA |
| *Zm*_XM_008654778 (*Odc3*)-qR | TCTCTGGCAGAAGGTACCCG |
| *Zm*_NM_001323076 (*Adc1*)-qF | GCTACGGCTCAAGGTACCAG |
| *Zm*_NM_001323076 (*Adc1*)-qR | CCGAACTCCACAATGTCCTC |
| *Zm*_NM_001138726 (*Adc2*)-qF | GTTCCCCATGATCCTTCGCT |
| *Zm*_NM_001138726 (*Adc2*)-qR | CCAGGTTGCAGTAGATGCCA |
| *Zm*_XM_023300699 (*Adc3*)-qF | CCAGATGTTGCTCTCCTTCAACT |
| *Zm*_XM_023300699 (*Adc3*)-qR | AAAAGTACCAACGGCGGCGA |
| *Zm*_XM_008671538 (*Adc4*)-qF | TGTCCTCCTGCTCAGAACCG |
| *Zm*_XM_008671538 (*Adc4*)-qR | AACTGGCGGGTTCCAGAATA |
| *Zm*_NM_001156222 (*Samdc1*)-qF | GCCTTCCCCACACAAGAACT |
| *Zm*_NM_001156222 (*Samdc1*)-qR | ACATAAGCATTGCCACCGGA |
| *Zm*_NM_001112243 (*Samdc2*)-qF | TTGCCAAAGAATTCCCTCCCC |
| *Zm*_NM_001112243 (*Samdc2*)-qR | TAGTACTCGGCCAGTTCCTCG |
| *Zm*_NM_001155794 (*Samdc3*)-qF | TGGGCTACCGGCAATTTGTT |
| *Zm*_NM_001155794 (*Samdc3*)-qR | TGCAGCTCACTATGGCAGAC |
| *Zm*_NM_001155467 (*Samdc4*)-qF | AGGTGGGATCCTCATCTACCA |
| *Zm*_NM_001155467 (*Samdc4*)-qR | ATTCTCGCCATCAAAGCAGC |
| *Zm*_NM_001155838 (*Spds1*)-qF | GCCAAGAGGGAGCTAGAAGC |
| *Zm*_NM_001155838 (*Spds1*)-qR | GCCGCAGTTAGCTTTTGTGG |
| *Zm*_NM_001147319 (*Spds2*)-qF | CCAACTCCAGAAGTCCTCCC |
| *Zm*_NM_001147319 (*Spds2*)-qR | CGAGGGAGGAACGGAGAAAG |
| *Zm*_NM_001155814 (*Spds3*)-qF | TGCCTGGTGCAGCTATGAAA |
| *Zm*_NM_001155814 (*Spds3*)-qR | GTGGAAGCGGAAACACAACC |
| *Zm*_NM_001112372 (*Spms1*)-qF | AAAGGGAGGTGCGGATGATG |
| *Zm*_NM_001112372 (*Spms1*)-qR | TATCATGGCAGTTGGCCTCG |
| *Zm*_XM_008664907 (*Spms2*)-qF | GTCGTTGAGTTGGACCCCTT |
| *Zm*_XM_008664907 (*Spms2*)-qR | TGCCATCTCCCAAATGGACC |
| *Zm*_NM_001111636 (*Pao1*)-qF | GAAAGTATGACTAGGAAGCTACAG |
| *Zm*_NM_001111636 (*Pao1*)-qR | GAATAATGAAAAGGCATATGCCGC |
| *Zm*_NM_001323613 (*Pao2*)-qF | CGGCTCTAAAAGCAGACGAG |
| *Zm*_NM_001323613 (*Pao2*)-qR | CAAGTAACGCCAGGCACATG |
| *Zm*_NM_001329439 (*Pao3*)-qF | CTAAGCAGACGAGAGGTTGTC |
| *Zm*_NM_001329439 (*Pao3*)-qR | GCACATTCCTCATGCCGAAG |
| *Zm*_NM_001176693 (*Pao4*)-qF | GATATCCAGGTTGTAAGAGAGAATC |
| *Zm*_NM_001176693 (*Pao4*)-qR | CTGACGAAAAACAGCATCCACC |
| *Zm*_NM_001175862 (*Pao5*)-qF | CTTAGGTTGCAGACTTGCAG |
| *Zm*_NM_001175862 (*Pao5*)-qR | CCAAATCCATATACCTTAGCAGT |
| *Zm*_NM_001137032 (*Pao6*)-qF | AGGAATGCAGGAAGCGGCTC |
| *Zm*_NM_001137032 (*Pao6*)-qR | GTGATCCAATCTCACTCAGGT |
| *Zm*_NM_001158589 (*Rib*)-qF | GGCTTGGCTTAAAGGAAGGT |
| *Zm*_NM_001158589 (*Rib*)-qR | TCAGTCCAACTTCCAGAATGG |


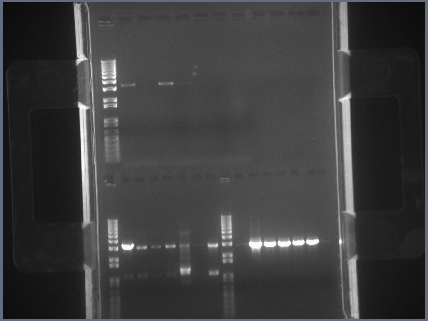


1 2 3 4 5 6 Con

2.0 kb

3.0 kb

1.0 kb

**B**


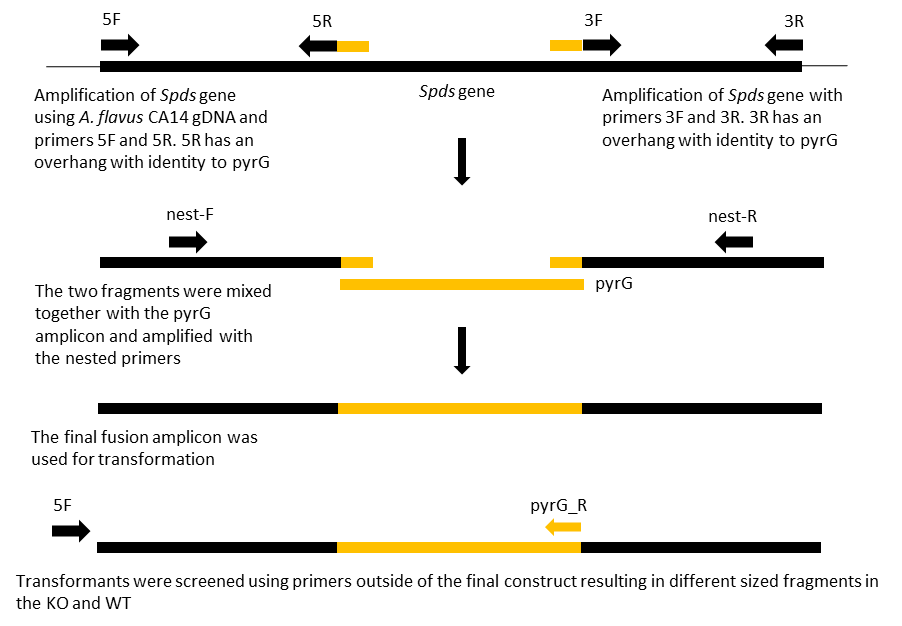


**A**

**C**


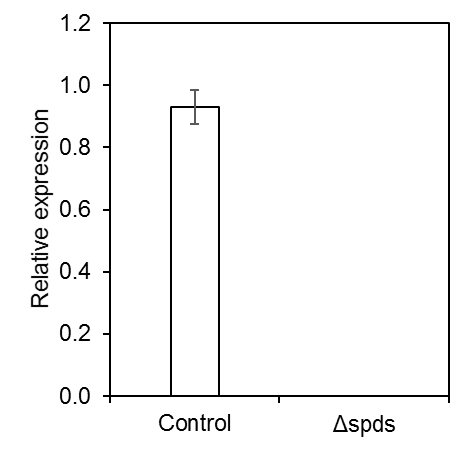


**Figure S1. Analysis of putative *A. flavus* CA14 *Δspds* transformants. (**A) Schematic representation of *Spds* gene knockout in *A. flavus* CA14 strain; PCR confirmation of *A. flavus* (CA14 strain) *Δspds* mutants and *Spds* expression in the control (CA14 gDNA) vs. *Δspds* mutant; (B) PCR amplification of gDNA isolated from *Δspds* mutant and CA14 gDNA (control) using *Spds*_5F and *pyrG*_R, shows a diagnostic band size of ~3 kb in the *Δspds* mutant only (green color numbers denote correct colonies); and (C) qRT-PCR of selected *Δspds* gene knockout mutant confirming inactivation of *Spds* expression. RNA was isolated from CA14 control and *Δspds* mutant after 48 h static growth on CZ+AS (ammonium sulfate) broth in the dark at 30°C. The relative gene expression level was normalized to the *A. flavus β-tubulin* gene expression.


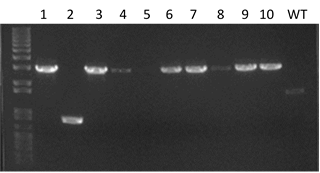


3.0 kb

2.0 kb

2.0 kb

1 2 3 4 5 6 7 8 9 10 Con

**B**


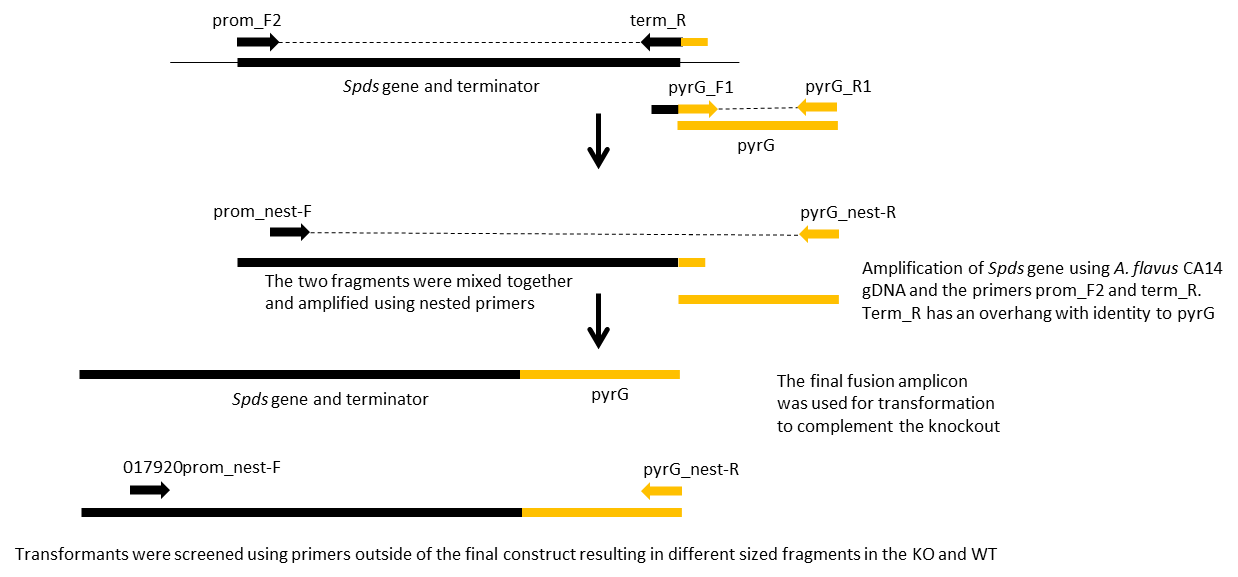


**A**

**C**


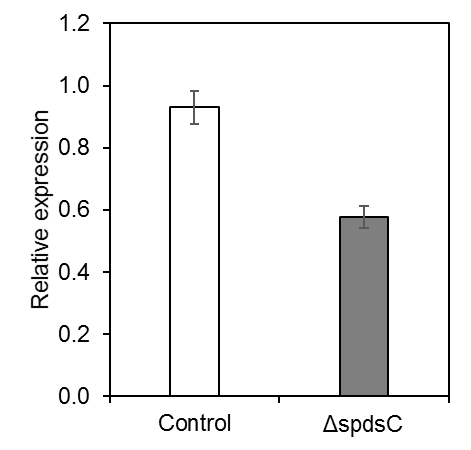


**Figure S2. Analysis of genetically complemented putative *A. flavus* CA14 *Δspds^C^* transformants.** (A) Schematic representation of the integration of *pyrG-Spds* PCR product at the site of the *Spds* deletion (via homologous recombination) and predicted PCR product sizes using diagnostic primer pairs; (B) PCR amplification of gDNA isolated from *Δspds*^c^ and CA14 gDNA (as control) using 017920prom_F2 and *pyrG*_R1 primers (Table S1), shows a diagnostic band size of 2.8 kb if integrated at the site of the gene in the *Δspds*^c^ strain only (green color numbers denote correct colonies); and (C) qRT-PCR of selected *Δspds^C^* complementation strain confirming expression of *Spds*. RNA was isolated from CA14 control and *Δspds^C^* mutant after 48 h static growth on CZ+AS (ammonium sulfate) broth in the dark at 30°C. The relative gene expression level was normalized to the *A. flavus β-tubulin* gene expression.

**
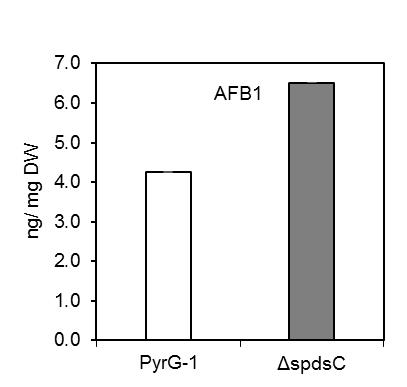
**

**Figure S3.** Aflatoxin B1 (AFB1) content in *A. flavus* CA14 control (PyrG-1) and *Δspds^C^* strain grown on solid A & M medium for 7 days in the dark at 30°C.
